# Arterial Embolism After Facial Fat Grafting: A Systematic Literature Review

**DOI:** 10.1007/s00266-023-03511-y

**Published:** 2023-08-10

**Authors:** Nicholas Moellhoff, Constanze Kuhlmann, Konstantin Frank, Bong-Sung Kim, Francesco Conte, Sebastian Cotofana, Nelson S. Piccolo, Norbert Pallua

**Affiliations:** 1 Division of Hand, Plastic and Aesthetic Surgery, University Hospital, LMU Munich, Ziemssenstraße 5, 80336 Munich, Germany; 2 Department of Plastic Surgery and Hand Surgery, University Hospital Zurich, Zürich, Switzerland; 3 Medical Faculty Rhenish, Westphalian Technical University, Aachen, Germany; 4 Pallua-Clinic Duesseldorf, Duesseldorf, Germany; 5 Department of Clinical Anatomy, Mayo Clinic College of Medicine and Science, Rochester, MN USA; 6 Division of Plastic Surgery, Pronto Socorro para Queimaduras, Goiânia, Brazil; 7 International Society of Plastic Regenerative Surgeons, Arlington Heights, IL USA

**Keywords:** Lipografting, Facelift, Facial plastic surgery, Liposuction, Arterial embolism, Vascular complication

## Abstract

**Background:**

While autologous fat grafting of the face is considered a generally safe procedure, severe complications such as arterial embolism (AE) have been reported.

**Objective:**

To summarize data on injection-related visual compromise, stroke, and death caused by arterial embolism after facial fat transplantation.

**Materials and Methods:**

Plastic surgery societies were contacted for reports on AE after autologous facial fat injection. In addition, a systematic literature review was performed. Data extracted included study design, injection site/technique, symptoms, management, outcome, and etiology.

**Results:**

61 patients with a mean age of 33.56 ± 11.45 years were reported. Injections targeted the glabella or multiple facial regions (both *n* = 16/61, 26.2%) most commonly, followed by injections in the temples (*n* = 10/61, 16.4%) and the forehead (*n *= 9/61, 14.8%). The mean volume injected was 21.5 ± 21.5 ml. Visual symptoms were described most frequently (*n* = 24/58, 41.4%) followed by neurological symptoms (*n* = 20/58, 34.5%), or both (*n* = 13/58, 22.4%). Ophthalmic artery (OA, *n* = 26/60, 43.3%), anterior or middle cerebral artery (CA, *n* = 11/60, 18.3%) or both (*n* = 14/60, 23.3%) were most frequently occluded. Outcome analysis revealed permanent vision loss in all patients with OA occlusion (*n* = 26/26, 100%), neurological impairment in most patients with CA occlusion (*n* = 8/10, 80%), and vision loss in most patients suffering from both OA and CA occlusion (*n* = 7/11, 63.6%). Six patients died following embolisms.

**Conclusions:**

AE causes severe complications such as blindness, stroke, and death. Due to a lack of high-quality data, no evidence-based treatment algorithms exist. To increase patient safety, a database collecting cases and complications should be established.

**Level of Evidence III:**

This journal requires that authors assign a level of evidence to each article. For a full description of these Evidence-Based Medicine ratings, please refer to the Table of Contents or the online Instructions to Authors www.springer.com/00266.

## Introduction

A plethora of articles has been published focusing on complications after minimally invasive procedures for rejuvenation of the face, amelioration of facial wrinkles, contouring and augmentation of facial features [1–4]. One of the most devastating complications, i.e., injection-related visual compromise (IRVC) has gained attention, due to increasing reports of blindness after soft-tissue filler injections [5, 6]. The increase in IRVCs can be linked to the constant rise of procedures in the facial region over the past decade. This is accompanied by an improved understanding of the underlying pathophysiology [7]. Retrograde intravascular administration of a bolus of soft-tissue filler can cause occlusion of the ophthalmic/central retinal/posterior ciliary arteries, irrespective of the physicochemical properties of the injected material. Several treatment algorithms have been established, providing protocols for the management of these complications [8, 9]. Exemplary, these protocols provide specific recommendations including retrobulbar hyaluronidase injections and pharmacotherapy in case of an IRVC caused by a hyaluronic acid (HA)-based filler.

As HA-based fillers are categorized as medical devices, strict regulations and rigorous post market surveillance are imposed by respective authorities, *e.g.,* the Food and Drug Administration (FDA) in the USA or the European Medicines Agency (EMA) in Europe. Databases such as the Manufacturer and User Facility Device Experience (MAUDE) database collect post-marketing safety surveillance data of (serious) adverse events for FDA-approved medical devices [10]. While most studies focus on arterial embolism (AE) after HA-based soft-tissue filler injections, there are also reports describing cases of blindness and even stroke after autologous fat injections in the face. According to the International Society of Aesthetic Plastic Surgeons (ISAPS), autologous fat grafting of the face was ranked as the 7 most common aesthetic surgical procedure worldwide with a total of 589,494 procedures performed [11]. Yet, as autologous fat is not classified as a medical device or drug, pharmacovigilance measures including databases on adverse events and complications of this procedure are necessary. In contrary to HA injections, the absence of effective antidotes to arterial fat embolisms results in a lack of specific recommendations and treatment strategies. As the autologous fat transfer is increasingly being performed by healthcare providers without specific training in plastic and aesthetic surgery, there is need for increasing awareness regarding AE after autologous fat injection of the face.

This systematic literature review aims to summarize current data on the topic and analyze the etiology, facial regions injected, injection techniques, and management of cases where visual and neurological complications occurred. In addition, the aim was to investigate whether and how complications after autologous fat injections in the face are recorded and documented, on both national and international level.

## Materials and Methods

A total of 16 national and international plastic surgical societies were contacted via publicly available contact details (i.e., contact email addresses provided on the respective websites) and questioned whether a database monitoring complications following autologous fat injection of the face exist. (Table 1) In addition, a systematic literature review was performed according to the Preferred Reporting Systems for Systematic Reviews and Meta-Analysis (PRISMA) guidelines [12]. PubMed and Embase databases were searched for manuscripts on AE after autologous facial fat grafting. The following search strategies were utilized:

1. PubMed: (((((autologous fat transplantation[MeSH Terms]) OR (fat injection[MeSH Terms])) OR (fat grafting[MeSH Terms])) AND (face[MeSH Terms])) OR (facial[MeSH Terms])) AND (embolism[MeSH Terms]), Most Recent,, “(((((( ““autolog”” [All Fields] OR ““autologous”” [All Fields] OR ““autologic”” [All Fields] OR ““autological”” [All Fields] OR ““autologous”” [All Fields] OR ““autologously”” [All Fields]) AND ““fat”” [All Fields]) AND ““transplantation”” [MeSH Terms]) OR (““fat”” [All Fields] AND ““injections”” [MeSH Terms]) OR (““fat”” [All Fields] AND ““transplantation”” [MeSH Terms])) AND ““face””[MeSH Terms]) OR ““face”” [MeSH Terms]) AND ““embolism”” [MeSH Terms]”
2. Embase: ((fat injection or fat transplantation or fat grafting or lipofilling) and (face or facial) and (blindness or vision loss or visual impairment or embolism))af.

**Table 1:** Overview of national and international societies contacted questioning the existence of a database monitoring complications after autologous fat grafting of the face.

Society	Geographic region	Response	Database
ASPS	USA	✓	–
BAPRAS	UK	✓	No
DGPRÄC	Germany	✓	No
EASAPS	Europe	–	–
ESPRAS	Europe	–	–
EURAPS	Europe	–	–
IAAPS	India	–	–
ICOPLAST	International	–	–
ISAPS	International	✓	No
JSPRS	Japan	–	–
KSAPS	Korea	–	–
SBCP	Brazil	–	–
SECPRE	Spain	–	–
SICPRE	Italy	–	–
SOFCPRE	France	–	–
THPRS	Thailand	–	–

**Fig. 1:**
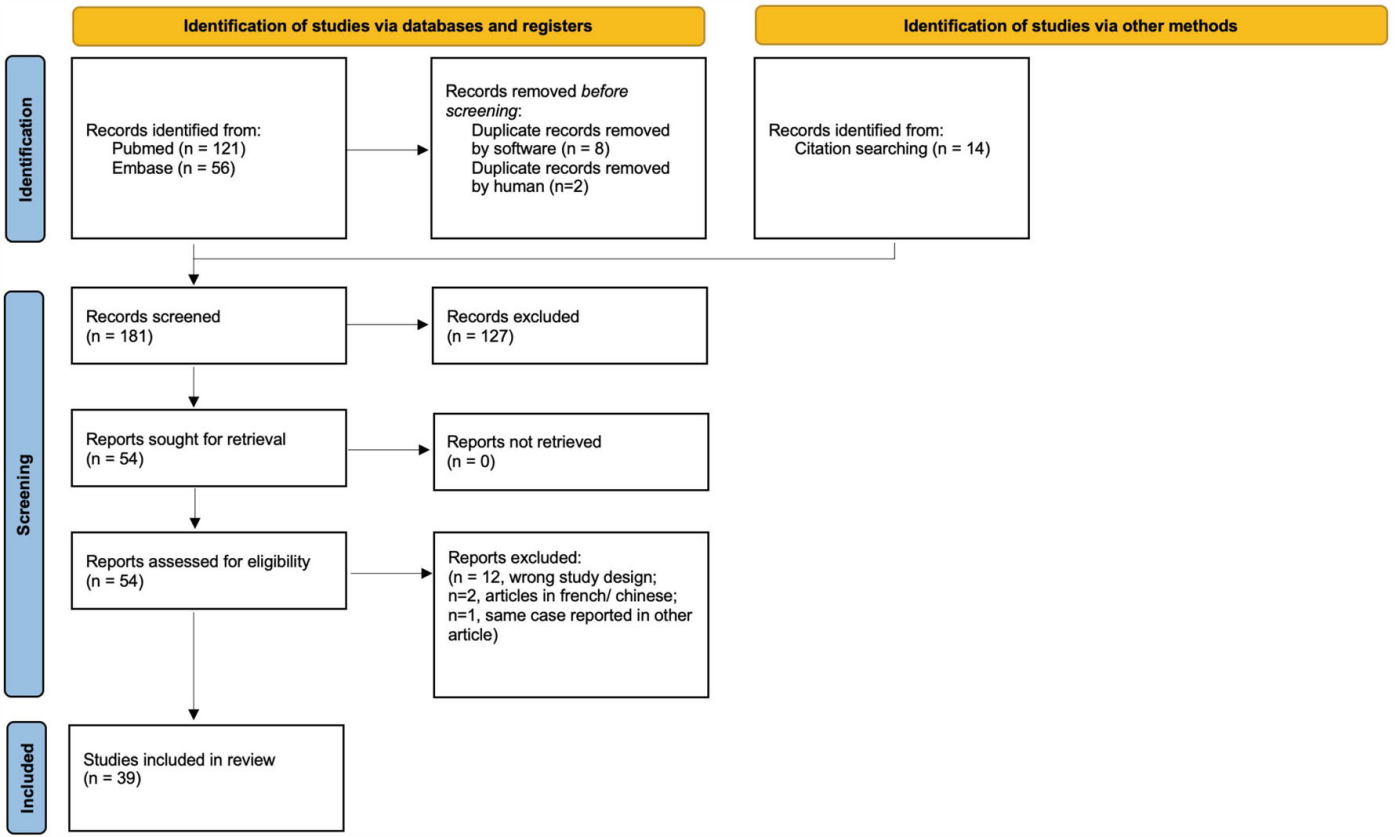
Detailed citation attrition diagram according to the PRISMA guidelines depicting the search strategy.

All cases of AE after injection of autologous fat in the face published in the English language were included, irrespective of patient age, gender, location, and technique of injection. Exclusion criteria were studies not performed in humans, injection of other fillers (i.e., hyaluronic acid, calcium hydroxyl apatite), or injection of fat outside of the face. Primary literature with original research articles was included, in addition to case reports and editorials, while secondary sources such as review articles and meta-analyses were screened, and reference lists were hand-searched to identify studies that may have been missed by the systematic literature search (snowballing). The literature search was completed on January 1st, 2023. Covidence systematic review software (Veritas Health Innovation, Melbourne, Australia) was utilized for the removal of duplicates, title and abstract screening, and full-text review. (Fig. 1) Two authors (N.M., C.K.) independently screened the titles and the abstracts for eligibility which were identified in the electronic database search.

### Data extraction

Data extracted included study title, first author, date, country, study design, gender, age, injection site, injection technique, volume injected, symptoms, the onset of symptoms, management, outcome, etiology, and follow-up time.

To enable statistical analysis, symptoms were categorized into neurological symptoms (including loss of consciousness, seizure, change of mental status, aphasia, paresthesia, paresis, headaches), visual symptoms (including vision loss, blindness, visual impairment, ocular pain), and cardiopulmonary symptoms (including respiratory, or cardiac decompensation, loss of oxygen saturation, dyspnea). Duration to the first onset of symptoms was categorized into the following ranges: immediate onset to 1 h post-injection, 2–23 h post-injection, and ≥ 24 h post-injection. Outcomes were categorized into vision loss, neurological impairment, and death.

Etiology was analyzed in cases where it was clearly described and identified. Categorization was performed as ophthalmic artery occlusion (OAO, including ophthalmic artery, central retinal artery, ciliary artery), cerebral artery occlusion (CAO, including anterior, middle, posterior cerebral artery), or both, pulmonary embolism, unclear or other.

### Statistical analysis

Data are presented as means with standard deviation, or as absolute and relative frequencies unless stated otherwise. Data were tabulated using Microsoft Excel (Microsoft Corporation, Redmond, Washington, USA). Descriptive statistics were performed using SPSS Statistics 28 (IBM, Armonk, NY, USA).

## Results

The follow-up and response rate of national and international plastic surgical societies contacted were low (Table 1). There were no reports on the existence of an established database monitoring complications following autologous fat grafting of the face, and therefore, no cases were obtained. Contrary, the systematic literature review yielded a total of 61 cases (*n* = 53/61, 86.9% females; *n* = 7/61, 11.5% males; *n* = 1/61, 1.6% transgender) with a mean age of 33.56 ± 11.45 (range 18–66) in 39 manuscripts (Table 2). The detailed citation attrition diagram can be found in Fig. 1. Most articles reported cases in China (*n* = 14/39, 35.9%) and South Korea (*n* = 12/39, 30.8%); however, reports were also found in other Asian countries, the US, Europe, Australia, and New Zealand. Most identified studies were case reports (*n* = 21/61, 53.8%), followed by case series (*n* = 9/61, 23.1%), letters to the editor (*n* = 4/61, 10.3%), case reports with reviews (*n* = 4/61, 10.3%), or correspondences (*n* = 1/61, 2.6%). The mean follow-up time was 166.73 ± 207.96 (range 3–730) days.

**Table 2:** Overview of studies on arterial embolism after facial fat grafting and detailed study information.

Refs.	First author	Year	Country	Study design	Gender	Age	Injection site*	Injection technique
[13]	Miao	2021	China	Case report	F	22	Temple	18-gauge needle
[14]	Gleeson	2011	UK	Case report	F	37	Cheek	18-gauge needle
[15]	Yoon	2003	Korea	Case report	F	39	Glabella	
[16]	Kang	2016	Korea	Case report	F	32	Glabella	18-gauge cannula
[17]	Feinendegen	1998	Switzerland	Case report series	M	45	Multiple	2 mm cannula
					F	47	Periorbital	
[18]	Wang	2018	China	Case-report series	F	30	Temple	
					F	22	Temple	
[19]	Teimourian	1988	USA	Letter to the Editor	F	45	Glabella	
[20]	Liu	2021	China	Case-report series and review	F	18	Temple	
					F	19	Frontal	
[21]	Danesh-Meyer	2001	New Zealand	Case-report	M	43	Multiple	Large-bore cannula
[22]	Hong	2014	Korea	Case-report series	F	27	Multiple	
[23]	Han	2006	Korea	Case-report	F	39	Multiple	
[24]	Huo	2018	China	Case-report series	F	33	Glabella	
					F	25	Glabella	
					F	24	Periorbital	
					M	19	Glabella	
					F	25	Glabella	
					M	28	Glabella	
[25]	Thaunat	2004	France	Correspondence	M	39	Multiple	
[26]	Carle	2014	USA	Case-report series	F	Early sixties	Forehead	
[27]	Liu	2020	China	Case-report	F	35	Multiple	
[28]	Dhooghe	2022	Belgium	Case-report and review	D	57	Multiple	
[29]	Chen	2014	China	Case-report series	F	24	Temple	0.3 mm needle
					F	47	Forehead	1.2 mm needle
					F	24	Multiple	
					F	36	Multiple	23-gauge needle
					F	33	Multiple	12-gauge needle
					F	27	Multiple	1 mm needle
					F	27	Temple	2 mm needle
[30]	Park	2012	Korea	Case-report series	F	66	Glabella	
					F	40	Nasolabial fold	
					F	18	Nasolabial fold	
					F	24	Glabella	
					F	37	Glabella	
					F	19	Glabella	
					F	26	Glabella	
[31]	Wang	2014	China	Case report	F	22	Multiple	2 mm cannula
[32]	Liu	2019	China	Case report	F	42	Temple	
[33]	Lee	2012	Korea	Case report	F	26	Multiple	
[34]	Shen	2016	China	Case report	F	30	Multiple	
[35]	Qian	2021	China	Case report and review	F	28	Multiple	
[36]	Egido	1993	Spain	Letter to the Editor	F	47	Glabella	
[37]	Hu	2011	China	Letter to the Editor	F	28	Temple	
[38]	Roshandel	2015	Australia	Case report	F	65	Forehead	
[39]	Lee	2010	Korea	Case report	F	24	Forehead	
[40]	Szantyr	2017	Poland	Case report and review	M	38	Multiple	20-gauge cannula
[41]	Lee	2011	Korea	Case report	F	44	Periorbital	
[42]	Liu	2020	China	Case-report series	F	29	Forehead	20-gauge needle
					F	46	Forehead	20-gauge needle
					F	38	Forehead	20-gauge needle
[43]	Xing	2012	Canada	Case report	F	23	Nose	
[44]	Park	2011	Korea	Case report	F	39	Nose	
[45]	Lu	2013	China	Case report	M	22	Temple	
[46]	Dreizen	1989	USA	Case report	F	44	Glabella	
[47]	Park	2008	Korea	Case report	F	27	Nasolabial fold	
[48]	Lee	1996	Korea	Letter to the Editor	F	42	Nasolabial fold	
[49]	Hong	2014	Korea	Case report	F	31	Glabella	
[50]	Putthirangsiwong	2022	Thailand	Case-report series	F	32	Forehead	
[51]	Zhou	2019	China	Case report	F	22	Temple	2.5 mm cannula

Refs.	Volume	Symptoms	Onset	Management	Outcome	Etiology	FU
[13]	20 ml per side	Unconsciousness	3 h	Decompressive hemicraniectomy, intravenous mannitol, vasoactive drugs, ceftriaxone, methylprednisolone	Reduced motor function, decreased sensation, hemianopsia	Fat embolism MCA	
[14]	35 ml per side	Hypoxic respiratory failure and cardiovascular decompensation	I	Invasive ventilatory support and an adrenaline infusion and was transferred to the critical care unit for organ support	Death	Cardiorespiratory failure secondary to fat emboli to the lung arterioles	Death
[15]	5 ml	Mental change, aphasia, and right hemiplegia	I	Decompressive hemicraniectomy	Death	Unclear, probably direct intravasation of fatty particles into the CA system, infarction left hemisphere	Death
[16]		Global aphasia and right complete sensorimotor hemiplegia with stuporous mentality		Thrombolysis	Loss of vision on the left eye, reduced motor function in the extremities	OAO, Hyperacute and embolic infarctions MCA and ACA territory	3 months
[17]		Global aphasia, mild right hemiparesis	7 h		Aphasia	Infarction CRA and temporoparietal region of the MCA through fat embolism	10 months
		Left eye pain, unresponsiveness, stupor, hemiplegia, global aphasia	I		Loss of vision on the left eye, regained ability to walk, improvement of the aphasia	OAO infarction of the left hemisphere	Few weeks
[18]		Left limb weakness		Decompressive hemicraniectomy, multiple treatments	Paralysis of the left limb	Brain infarction through fat embolism	
		Unconsciousness, left limb weakness		Decompressive hemicraniectomy, multiple treatments	Bilateral loss of vision, motor function impairment	CRAO	2 years
[19]		Unilateral loss of vision and pain on the right eye	I	Supportive treatment	Loss of vision on the right eye	OAO	
[20]		Unconsciousness, left hemiplegia, and vomiting	24 h	Decompressive hemicraniectomy, multiple treatments	Death	Occlusion of ECA, respiratory failure	Death
		Unilateral loss of vision, hemiplegia	4 h		Loss of vision on the left eye	Multiple retinal arterioles were occluded	3 months
[21]	3 ml on each side, 3 ml on both lips	Eye and head pain, disorientation, loss of vision, aphasia with right-sided hemiparesis.	10 min		Loss of vision on the left eye	Fat embolism MCA and OA	
[22]		Periocular pain, memory disturbance			Loss of vision on the left eye	CRAO, multiple small acute infarctions in the left frontal lobe	1 year
[23]		Unilateral loss of vision, headache	10 min			OAO with acute multiple infarcts in the territories of the bilateral ACA, MCA and PCA	
[24]		Motor disturbance in right extremities and loss of consciousness		Embolectomy + decompressive craniectomy	Motor aphasia, reduced motor function on right extremity	ICA and MCA M1 segment occlusion through fat embolism	
		Motor disturbance and loss of consciousness.		Embolectomy + decompressive craniectomy	Reduced motor function on right extremities	MCA M3 segment and ACA occlusion through embolism	
		Seizure accompanied with loss of consciousness and motor disturbances in left extremities.	2 h	Embolectomy	Death	ACA, ICA, MCA, M1 segment occlusion through fat embolism	Death
		Right hemiplegia, unconsciousness	1 h	Embolectomy + decompressive craniectomy	Aphasia, reduced motor function of the right extremities	MCA occlusion with fat embolism in the parietotemporal brain region. Massive brain infarct with severe brain edema.	
		Visual disturbance with no light sensation	I	Embolectomy	Decrease in visual acruity	OAO	
		Seizure accompanied with loss of consciousness	5 h	No treatment	Death		Death
[25]	17 ml	Confusion, hypertension	I	Reintubation and sedation	Moderately severe disability.	Ischemic lesions after fat embolism in ACA	1 year
[26]		Unilateral loss of vision, dysarthria	I		Loss of vision on the right eye	CRAO	
[27]		Left hemiplegia, left facial palsy	I	Oral aspirin 0,2 g once daily, atorvastatin 40 mg once daily p.o., and dexamethasone 10 mg i.v. once daily	Patient is able to walk	Fat embolism MCA	3 months
[28]			1 d		Death	Multiple microfat embolisms in the cerebral circulation, localized subarachnoid hemorrhages, and an intraventricular bleeding in the fourth ventricle	Death
[29]		Headache, ptosis, ophthalmoplegia		Nitroglycerin, digital massage, eye drops, aspirin, prednisone	Loss of vision on the right eye	OAO	90 days
	20 ml	Ptosis, ophthalmoplegia		Nitroglycerin, digital massage, eye drops, aspirin, prednisone	Loss of vision on the left eye	OAO	30 days
				Nitroglycerin, digital massage, eye drops, aspirin, prednisone	Loss of vision on the left eye	OAO	30 days
	2 ml	Ptosis, ophthalmoplegia		Nitroglycerin, digital massage, eye drops, aspirin, prednisone	Loss of vision on the right eye	OAO	36 days
	2 ml	Ptosis, ophthalmoplegia		Nitroglycerin, digital massage, eye drops, aspirin, prednisone	Loss of vision on the left eye	OAO	25 days
	5 ml	Ptosis, ophthalmoplegia, dizziness, vomiting		Nitroglycerin, digital massage, eye drops, aspirin, prednisone	Loss of vision on the right eye	OAO	11 days
	12 ml	Ptosis		Nitroglycerin, digital massage, eye drops, aspirin, prednisone	Loss of vision on the left eye	OAO	10 days
[30]		Ptosis, ophthalmoplegia	I	Intraarterial thrombolysis	Loss of vision on the left eye	OAO	5 days
		Ophthalmoplegia, exotropia	I	Intraarterial thrombolysis	Loss of vision on the left eye	OAO	511 days
		Ptosis, esotropia, ophthalmoplegia	I	Intraarterial thrombolysis	Loss of vision on the right eye	OAO	430 days
		Ptosis, exotropia, ophthalmoplegia	1 week		Loss of vision on the left eye	OAO, MCA infarction	63 days
		Ophthalmoplegia, exotropia	I	Anterior chamber paracentesis	Loss of vision on the right eye	OAO	3 days
		Exotropia	2 h	Anterior chamber paracentesis	Loss of vision on the left eye	OAO	40 days
			2 d	Anterior chamber paracentesis		ACA and MCA infarction through fat embolism, CRAO	16 days
[31]	25 ml frontal, 24 ml temporal	Right-sided hemiparesis (under anesthesia), aphasia, temporal necrosis	4-5 h	Decompressive hemicraniectomy, mannitol	Loss of vision on the left eye, aphasie	Occlusion of ICA, ECA, OA embolic infarction of ACA and MCA	2 months
[32]		Unresponsive, lethargic, global aphasia, right-sided hemiplegia	I	Decompressive hemicraniectomy, multiple treatments	Complete expressive aphasia and right-sided hemiplegia.	Fat embolism ICA, ACA, MCA	2 years
[33]		Unilateral loss of vision, hemiplegia	13 h	Methylprednisolone (9 mg/kg i.v.), prednisolone (30 mg/kg i.v.)		Two Episodes of Repeated Cerebral Fat Embolisms	
[34]	44 ml	Unconsciousness, left limb weakness	2 h	Supportive treatment, decompressive craniectomy	Severe neurological impairment	Extensive cerebral infarction through occlusion of the ICA and ECA through fat embolism	2 months
[35]	20 ml temple, 20 ml forehead, 17ml cheeks	Left limb movement disorder, unconsciousness	I	Craniectomy for decompression	Motor function impairment	Large cerebral infarction on the right frontal, temporal, and parietal lobes due to complete occlusion of her right ECA	4 months
[36]		Unilateral loss of vision, eye pain, left hemiplegia	I		Loss of vision on the right eye, reduced motor function in the left extremities, complete plegia of the right arm	Fat embolism to the MCA and OA	3 months
[37]		Aphasia, hemiparesis (right)	I	Mannitol, hydrocortisone, antiplatelet agents and hyperbaric oxygen therapy	Reduced motor function on right extremities	Occlusion of M1 segment of MCA	3 weeks
[38]		Unilateral loss of vision	Few h			Fat embolism of OA and MCA.	
[39]		Unilateral loss of vision, decreased sensation on the forehead and scalp, and paresthesias of the right leg	1 d	1 g/day methylprednisolone i.v. for 3 consecutive days	Loss of vision on the left eye	OAO with fat embolism infarction of the MCA	5 months
[40]	5 ml per region	Unilateral loss of vision, eye pain	I	Ocular massage, ocular drops: 0.5% timolol, brimonidine and dorzolamide to the right eye, 24 mg dexamethasone i.v., 500 ml of 20% mannitol i.v., 80 ml of 40% glycerol p.o., 500 mg of acetazolamide p.o.	Irregular visual field defects	OAO	2 years
[41]		Unilateral loss of vision, dysarthria, skin color of the nose changed to purple	I	Ocular massage, intravenous mannitolization, and oxygen and carbon dioxide therapy	Loss of vision on the left eye	OAO and MCA infarction	2 months
[42]	15 ml	Unilateral loss of vision, eye pain, weakness of right limb, nausea	I	Intravenous infusion of 500 mL of dextran glucose solution with 20 mg of dexamethasone and 250 mL of mannitol, neurotrophic factor therapy	Loss of vision on the left eye	OAO and left MCA infarction	3 months
	7 ml	Unilateral loss of vision, eye pain, nausea	I	Intravenous infusion of 10 mg of dexamethasone and energy mixture	Loss of vision on the left eye	OAO and ophthalmoplegia	3 months
	5 ml	Unilateral loss of vision, nausea, necrosis on forehead	I	Intravenous dexamethasone (10 mg) and local cold compression	Loss of vision on the left eye	OAO and avascular necrosis of the left forehead skin	3 months
[43]		Unilateral loss of vision	90 min	Medical treatment to lower eye pressure		OAO	
[44]		Unilateral loss of vision, eye pain	I	Pharmacomechanical thrombolysis	Loss of vision on the left eye	OAO	17 months
[45]		Unilateral loss of vision		High dose o vitamins for 1 month	Loss of vision on the left eye	CRAO	3 months
[46]		Hemicranial pain, and unilateral loss of vision	I		Loss of vision on the right eye	OAO	2.5 months
[47]		Unilateral loss of vision	10 min	Methylprednisolone 1 g/day/i.v for 3 days	Loss of vision on the right eye	CRAO	6 months
[48]	0.5 ml	Headache, dyspnoea, unconsciousness	I	Ocular massage, and oxygen and carbon dioxide therapy	No perception of light on the left eye	CRAO combined with brain infarction	3 months
[49]		Unilateral loss of vision, arm weakness, purple skin in the periocular region	I	Ocular massage, anterior chamber paracentesis, and volume expansion	Loss of vision on the right eye	Fat embolism of OA and MCA.	5 months
[50]	10 ml	Unilateral loss of vision, eye pain	I	Acetazolamide 500 mg p.o. followed by 250 mg every 6 h, 0.5% timolol eye and 0.15% brimonidine-P eye drops every 12 h, amoxicillin/clavulanic acid i.v. 1.2 g every 8 hours. Vascular recanalization via a transfemoral transvenous embolectomy, methylprednisolone 1000 mg i.v,, prednisolone p.o. 1 mg/kg/day for 2 weeks	Complete recovery	Superior Ophthalmic Vein Embolism	1 months
[51]	25 ml per side	Unconsciousness, left-sided hemiparesis	4 h	Mechanical lipectomy - Solitaire stent (4 × 20 mm) and Solumbra (continuously negative pressure attraction)	Mild motor function impairment in the left leg	Multiple fat embolisms in right ICA and MCA with basal ganglia ischemia	3 months

*I*, Immediately; ICA, Internal carotid artery; MCA, Middle cerebral artery; OA, Ophthalmic artery; CRA, Central retinal artery; ECA, External carotid artery; ACA, Anterior cerebral artery; PCA, Posterior cerebral artery; CRAO, Central retinal artery occlusion; OAO, Ophthalmic artery occlusion; I.v., intravenous; p.o., per os.

*Injection site “multiple“ includes all injections targeting more than one facial region, including “full-face“ approach or other not further specified injections.

### Injection

While injections were performed in all facial areas, a detailed analysis of the reported cases revealed that most frequently injections targeted the glabella region only or multiple facial regions (both *n* = 16/61, 26.2%), followed by injections in the temples (*n* = 10/61, 16.4%), the forehead (*n* = 9/61, 14.8%), the midface (including nasolabial fold and cheek, *n* = 5/61, 8.2%), the periorbital region (*n* = 3/61, 4.9%), and the nose (*n* = 2/61, 3.3%). The exact injection technique was reported infrequently. In those reports that included the injection technique, *n* = 11/17 (64.7%) reported the use of a needle, while *n* = 6/17 (35.3%) reported the use of a cannula during the injection. The mean total volume of injection (reported in *n* = 20 reports) was 21.5 ± 21.5 ml of autologous fat (range 0.5–70 ml).

### Onset of symptoms and outcome

The onset of symptoms ranged between immediate onset and 1 week postoperative. Most frequently, symptoms occurred immediately to 1 h post-injection (*n* = 33/50, 66.0%), followed by onset between 2 and 23 h post-injection (*n* = 12/50, 24.0%) and ≥ 24 h post-injection (*n* = 5/50, 10.0%). Visual symptoms were described most frequently (*n* = 24/58, 41.4%) followed by neurological symptoms (*n* = 20/58, 34.5%), or both visual and neurological symptoms (*n* = 13/58, 22.4%). Analysis of outcomes revealed permanent vision loss (*n* = 32/54, 59.3%), neurological impairment (*n* = 12/54, 22.2%), both neurological and visual impairment (*n* = 4/54, 7.4%), and death (*n* = 6/54, 11.1%).

### Management

Management of symptoms highly depended on the individual documented case and included conservative treatment (i.e., ocular massage), pharmacological (i.e., nitroglycerin, aspirin, prednisone, mannitol), minimally invasive (i.e., embolectomy), as well as operative interventions (i.e., decompressive hemicraniectomy).

### Etiology

**Fig. 2:**
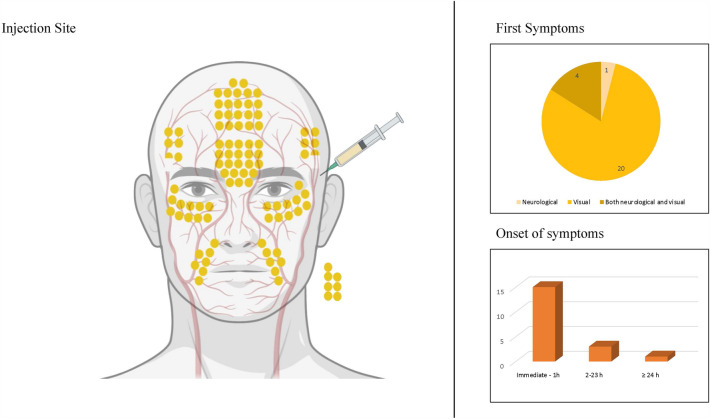
Analysis of injection site, first symptoms and onset of symptoms in patients with ophthalmic artery occlusion. Each dot on the left panel represents the injection site of 1% of cases. Dots outside of the face each represent 1% of cases where multiple (or not further specified) injection sites were targeted. Figure created with BioRender.com.

**Fig. 3:**
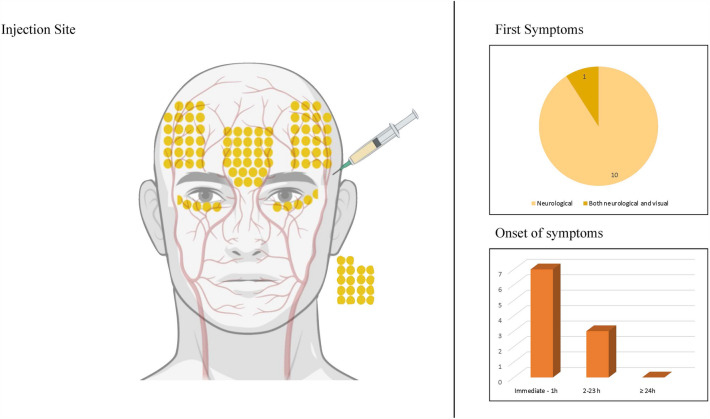
Analysis of injection site, first symptoms and onset of symptoms in patients with cerebral artery occlusion. Each dot on the left panel represents the injection site of 1% of cases. Dots outside of the face each represent 1% of cases where multiple (or not further specified) injection sites were targeted. Figure created with BioRender.com.

**Fig. 4:**
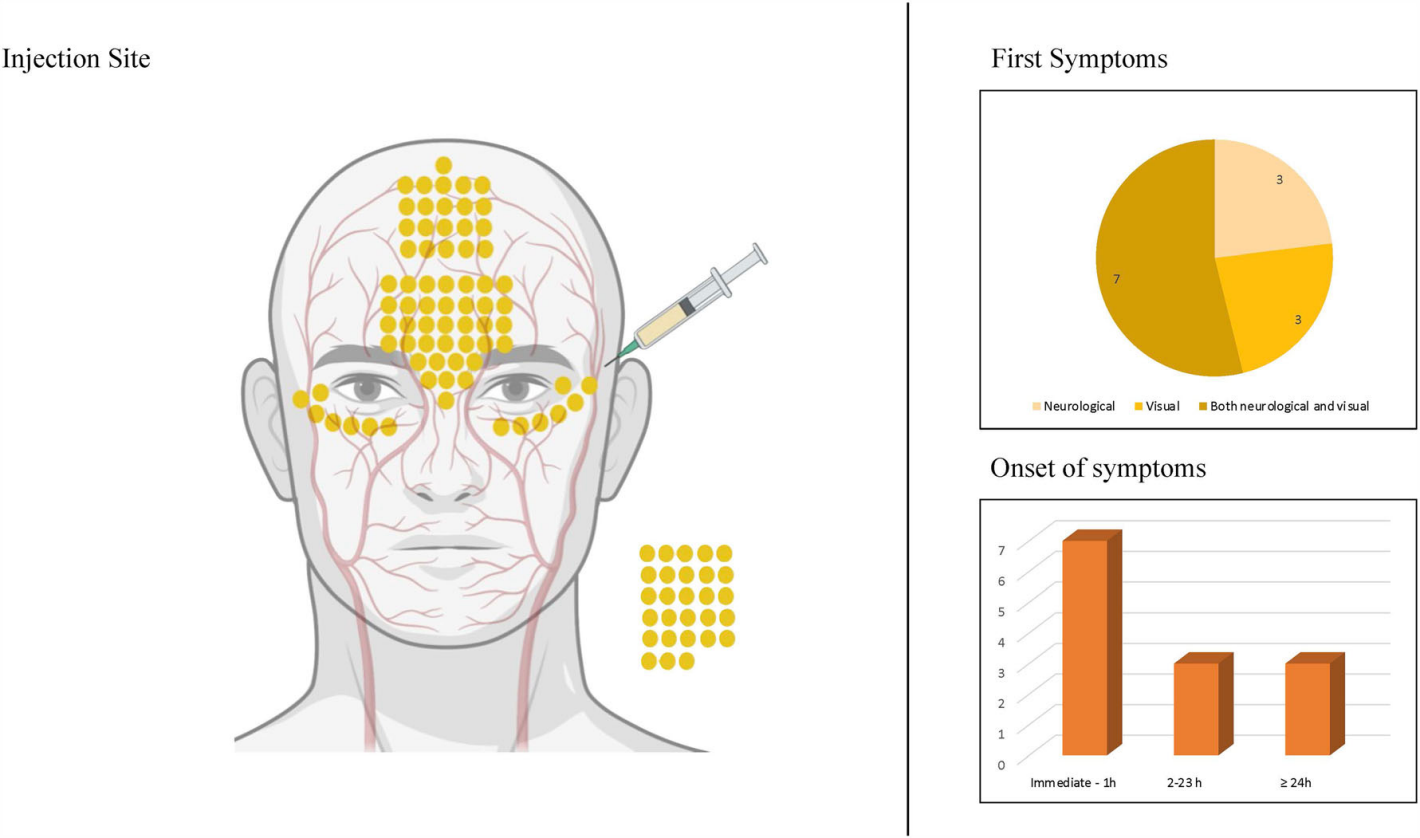
Analysis of injection site, first symptoms and onset of symptoms in patients with both ophthalmic artery occlusion and cerebral artery occlusion. Each dot on the left panel represents the injection site of 1% of cases. Dots outside of the face each represent 1% of cases where multiple (or not further specified) injection sites were targeted. Figure created with BioRender.com.

**Fig. 5:**
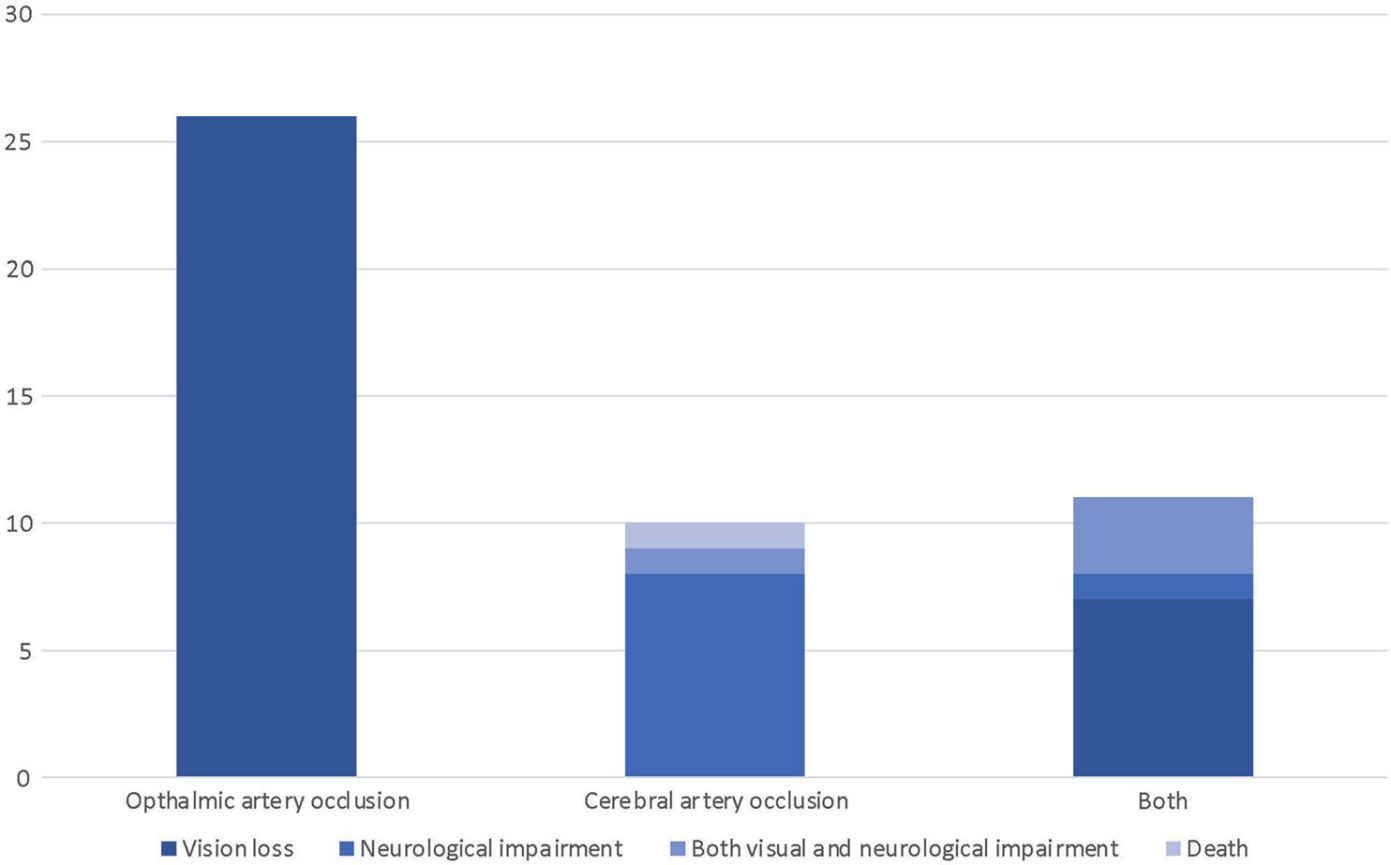
Analysis of outcomes with respect to the location of vascular occlusion.

Etiology was analyzed revealing OAO (*n* = 26/60, 43.3%), CAO (*n* = 11/60, 18.3%), or both (*n* = 14/60, 23.3%) to be the most frequent causes for the symptoms and outcomes described. Unclear/other causes and pulmonary embolism were reported in *n* = 8/60 (13.3%) and *n* = 1/60 (1.7%), respectively. Patients were stratified into three different groups, according to the etiology: OAO, CAO, and both OAO and CAO. The injection site, symptoms, the duration of the first symptoms, and outcomes were analyzed for each group (see Figs. 2, 3, 4 and 5). OAO occurred most frequently after injections into the glabella, forehead, and periorbital region (*n* = 6/26, 23.1% each), and CAO after injections into the temples (*n* = 5/11, 45.5%) and both OAO and CAO combined after injections into the glabella region (n = 5/14, 35.7%). Visual symptoms were experienced most frequently by patients within the OAO group (*n* = 20/26, 76.9%); neurological symptoms were reported most frequently by patients in the CAO group (*n* = 10/11, 90.9%) and a combination of visual and neurological symptoms by the group suffering from both OAO and CAO (*n* = 7/13, 53.8%). The most frequent onset of symptoms ranged between immediate-1h post-intervention in all groups. Outcome analysis revealed vision loss in all patients with OAO (*n* = 26/26, 100%), neurological impairment in most patients with CAO (*n* = 8/10, 80%), and vision loss in most patients suffering from both OAO and CAO (*n* = 7/11, 63.6%). Deaths were not associated primarily with a specific etiology, or injection site (Table 3).

**Table 3:** Overview of deaths with regard to etiology and injection site.

Death ID	Injection site	Etiology
1	Cheek	Cardiorespiratory failure secondary to fat emboli to the lung arterioles
2	Glabella	Unclear, probably direct intravasation of fatty particles into the CA system
3	Temple	Occlusion of ECA, respiratory failure
4	Periorbital	ACA, ICA, MCA, M1 segment occlusion through fat embolism
5	Glabella	–
6	Multiple	Multiple microfat embolisms in the cerebral circulation, localized subarachnoid hemorrhages, and an intraventricular bleeding in the fourth ventricle

ACA, Anterior cerebral artery; ECA, External carotid artery; ICA, Internal carotid artery; MCA, Middle cerebral artery

## Discussion

This systematic review revealed a paucity of high-quality data on AE after facial autologous fat injections. Incidence, clinical manifestation, recommendations and or algorithms for treatment, and advice regarding the prevention of arterial embolization are based on case reports, case series, letters, and correspondences. Contacting national and international societies revealed no centralized and standardized monitoring of adverse events after autologous fat grafting of the face. Consequences of AE after autologous fat injection are, however, undeniable. The data retrieved by the systematic literature review revealed severe visual and neurological deficits which most commonly occur within a few seconds to minutes after intraarterial injection. These include vision loss, stroke-related symptoms such as hemiplegia, and even death, depending on the location of the fat embolus and the artery occluded. Hence, an understanding of the pathomechanisms, the symptoms, the potential etiology, and outcomes is essential for surgeons to recognize, manage, and prevent these complications accordingly.

### Vascular anatomy and pathophysiology

The face is supplied by the internal and external carotid artery (ICA, ECA), which give off major branches to the upper, middle, and lower face [52]. These branches form a vast anastomotic network between each other and between both hemifaces. Retrograde injection of autologous fat into the ICA system can cause severe complications, depending on the exact location of the vascular occlusion. Two mechanisms are possible, i.e., direct intravasation and retrograde bolus advancement of fat into branches of the ICA system (such as the dorsal nasal, the supratrochlear, or supraorbital artery in the glabella region) or by injecting into the ECA system with consecutive embolus occlusion of the internal system via one of the many anastomoses between external and internal carotid artery (i.e., supraorbital arcade which marks anastomoses of the superficial temporal, transverse facial, and zygomatic orbital, as well as supraorbital/supratrochlear arteries) [7, 53, 54]. Hence, prior to autologous fat grafting in the face, surgeons must be aware of the three-dimensional course of vessels, to adapt the respective tissue layer, depth, and direction of injection.

The glabella, forehead, and temple region were among the sites most frequently affected by injection-related AEs. In addition, periorbital and nasolabial injections belong to the most popular facial regions in which autologous fat injection is being performed. Hence, the arterial vasculature of these facial regions shall be further highlighted.

The glabella region is likely to be most prone to IRVC and/or stroke, as terminal branches of the ophthalmic artery (ICA vascular network) provide the vascular supply to this region [5, 6]. The supratrochlear, the supraorbital, and the dorsal nasal artery branch off the ophthalmic artery and exit the bony skull via distinct foramina/notches. The supraorbital and supratrochlear arteries emerge from the supraciliary arch of the frontal bone and course cranially toward the forehead. In the glabella and lower forehead, they are located in a deep layer beneath the frontalis muscle. During their course cranially, branches of these arteries can change plane to travel more superficially, above the frontalis muscle, at approximately 1.5–2.5 cm superior to the supraorbital rim [55, 56]. However, it needs to be pointed out that the supratrochlear, as well as the supraorbital artery often branch off into a superficial and a deep branch. The superficial branch might emerge to the superficial layer much more caudal, than the deep branch. The dorsal nasal artery runs inferiorly toward the nose 4–5 mm lateral to the midline [57]. In most cases, it can be found superficially, within the subcutaneous tissue on top of the nasalis muscle [58]. A rich anastomotic network with the ECA can be found, as it has multiple anastomoses with the angular artery and the lateral nasal artery, the palpebral arteries, the infraorbital artery, and the superior labial artery [59, 60].

The superficial and deep temporal arteries provide vascular supply to the temple region. The superficial temporal artery branches off the ECA at the anterior border of the tragus and courses obliquely toward the temporal crest. It is located within the superficial temporal fascia [52, 61, 62]. The deep temporal arteries branch off the maxillary artery, which itself is a branch of the ECA. The anterior and posterior deep temporal artery are located beneath the temporalis muscle, approximately 2.5 cm lateral to the lateral orbital rim; however, this landmark should not be considered as marking a safe zone, as perforators of the anterior deep temporal artery might course closer to the lateral orbital rim [63].

Recent data revealed up to five anastomotic pathways between the ICA and ECA in the upper face (including temples, forehead, and the periorbital region). These are formed via the frontal branch of the superficial temporal artery, branches of the transverse facial artery, and the zygomatic-orbital artery with terminal branches of the ICA both deep to and superficial to the superficial fascia (i.e., the frontalis muscle in the forehead, the superficial temporal fascia in the temple, and the orbicularis oculi muscle in the periorbital region) [54]. These findings provide an explanatory model as to why IRVC and stroke can occur after injections of the temple, the zygomatic arch, and the lateral periorbital region.

### Management

No standardized guidelines for the management of AE after autologous fat grafting of the face exist. Unlike HA injections which can be dissolved using hyaluronidase, there is no agent for fat emboli resolution of comparable effect. Management of AE after facial fat grafting, therefore, consists of conservative therapy, pharmacotherapy, minimally invasive interventions, and operative procedures. Most cases of vision loss in patients with OAO were managed using symptomatic treatment via digital ocular massage, eye drops, aspirin, prednisone, and nitroglycerin. Unfortunately, treatment modality had no effect on the outcome as all patients with OAO suffered from irreversible vision loss. Occlusion of the ophthalmic, central retinal, or ciliary arteries causes ischemia of the retina. The retinal cells are especially susceptible to hypoxia and vascular occlusion thus leading to vision loss within minutes rather than hours [64]. To date, no effective intervention to reverse retinal hypoxia and cell degradation exists. Emergent thrombolytic therapy is being explored; however, further randomized clinical trials are warranted [65]. It needs to be pointed out that treatment approaches aiming to improve retinal perfusion (i.e., carbogen, acetazolamide, topical beta-blockers, ocular massage, and anterior chamber paracentesis) in the setting of a central retinal artery occlusion caused by a stroke lack efficacy so far, which furthermore highlights the unfortunate therapeutic situation for now [66–68].

Patients with neurological impairment and stroke after CAO treatment of choice was mechanical embolectomy and/or decompressive hemicraniectomy in combination with systemic corticosteroid treatment and/or thrombolysis in most cases. However, once more the outcome was highly unfavorable despite these treatment efforts. Due to the limited effectiveness of treatment of vascular occlusion, preventive measures appear to be the more dominant solution within the conceptual management of this complication entity.

### Prevention

Several measures to prevent vascular occlusion after soft-tissue filler injection have been defined [69–72]. General measures such as slow retrograde injections of small aliquots, pre-injection aspiration, low injection pressure, and cannula use are advisable. However, these recommendations are often eminence, rather than evidence-based, and the applicability of these preventive measures for fat grafting can be limited. For HA-based soft-tissue filler injections, boluses smaller than 0.1cc are often recommended to avoid IRVC, as the diameter of the ophthalmic artery was reported to range between 0.04 and 0.12 ml [73]. Injection of such small fat boluses may be considered neither practical nor effective, which limits this preventive measure in the context of fat grafting. With the emergence of micro-, nano–fat and lipoconcentrate [74], it needs to be assessed whether different reconstitutions of fat do possess different thrombogenic properties and carry eventually different risks of arterial occlusion.

Previously published data revealed that the force required for arterial penetration increases with increasing needle/cannula size, highlighting that larger needles/cannulas are safer, compared to ones with a smaller diameter [75]. At the same time, cannulas are considered safer than needles [76] as the force required for vascular wall penetration is significantly increased when using blunt-tip cannulas, when compared to needles [75]. In line with this, and although reporting was limited to merely a few cases, the incidence of AE after fat injection was more frequently observed using needles than cannulas in this systematic review. Schiraldi et al. summarized important safety measures based on their systematic review of complications after facial fat grafting including slow, retrograde injection of small aliquots using large diameter cannulas (i.e., 18 Gauge or larger) and small (1ml) syringes [77]. Foremost, it is the opinion of the authors of this manuscript that anatomical knowledge and understanding of the underlying vasculature and its 3-dimensional course are paramount to increase the safety prior to performing these procedures. As for hyaluronic acid injections, the feasibility of ultrasound guided injection of fat needs to be elaborated in the future [78, 79].

### Reporting and Monitoring of Complications

To the knowledge of the authors, and after contacting several plastic surgery societies, no standardized and centralized database collecting complications after facial augmentation using autologous fat exists. Given the low incidence of AE after autologous fat injection—preventing the conduct of meaningful clinical trials—paired with the severity of outcomes, there is a strong need for documentation of this complication cluster to create evidence-based prevention/management algorithms. Importantly, data acquisition should be governed by a centrally maintained but universally accessible entity and should encompass high resolution data including precise details of the entire periprocedural phase plus sufficient follow-up. Unfortunately, lipografting of the face—like hyaluronic acid injection—can be performed by medical professionals who are not specialized within the field of plastic, reconstructive and aesthetic surgery in some countries. Most cases in literature were reported in China (*n* = 29/61, 47.5%) and Korea (*n* = 18/61, 29.5%). Although there is a lack of reliable data to quantify the number of facial fat grafting procedures performed annually in these countries, the demand for aesthetic surgery is known to be high [80]. Recently, reports have uncovered so called *ghost-surgeries* [81], performed by un- or underqualified practitioners, and while there remains room for speculation, this could provide an explanation for an increase in complications.

Importantly, the patients themselves are often unaware of the qualification of the medical professional treating them, as the term aesthetic or beauty surgeon is often not protected by law and is in many countries not limited to specialized plastic (and aesthetic) surgeons [82, 83]. Thus, there needs to be a change and international harmonization in legislation to prevent complications arising from use of wrong injection technique, plane, and material by unqualified practitioners.

## Conclusions

Severe complications such as blindness, stroke, and death can occur due to AE after facial fat grafting. Based on currently available literature, 61 cases were identified, with approximately 600.000 procedures being performed annually [11]. While these complications can therefore be considered rare, exact numbers of incidence cannot be provided due to the limited data available. In addition, there is reason to believe in underreporting of these complications. Due to the severity of these complications, in a supposedly simple procedure such as lipografting of the face, this article wants to sensitize the audience toward them. Presently, the dearth of high-quality data inherently leads to missing evidence-based prevention/treatment algorithms. To increase patient safety and awareness for the severe complications and outcomes associated with AE after facial fat injection, an international database for documentation and surveillance of fat grafting-induced complications is pivotal.
